# Burdens of Postoperative Infection in Endoscopic Retrograde Cholangiopancreatography Inpatients

**DOI:** 10.7759/cureus.5237

**Published:** 2019-07-25

**Authors:** Shanthini Kuduva Rajan, Sowmya Madireddy, Paul Rahul Jaladi, Virendrasinh Ravat, Anum Masroor, Uwandu Queeneth, Wahida Rashid, Rikinkumar S Patel

**Affiliations:** 1 Internal Medicine, Tirunelveli Medical College, Tirunelveli, IND; 2 Internal Medicine, Mamata Medical College, Khammam, IND; 3 Internal Medicine, Rajiv Gandhi Institute of Medical Sciences, Kadapa, IND; 4 Internal Medicine, Larkin Community Hospital, South Miami, USA; 5 Medicine, Khyber Medical College, Khyber Pakhtunkhwa, PAK; 6 Medicine, Maastricht University, Maastricht, NLD; 7 Internal Medicine, Dhaka Medical College, Dhaka, BGD; 8 Psychiatry, Griffin Memorial Hospital, Norman, USA

**Keywords:** ercp, post operative infection, hospitalization, healthcare cost, endoscopic retrograde cholangiopancreatography

## Abstract

Objectives

Our objective in this study is, firstly, to determine postoperative (POI) rates in endoscopic retrograde cholangiopancreatography (ERCP) procedures stratified by patients’ demographic and hospitals’ characteristics in the United States, and secondly, to evaluate the demographic and comorbid risk factors associated with POI in ERCP inpatients, as well as its impact on the length of stay (LOS) and total charges.

Methods

The total sample of 28,525 inpatients with a principal procedure of ERCP from the Nationwide Inpatient Sample (NIS) was included and grouped by co-diagnosis of POI (N=300, 1.05%). We used a logistic regression model and descriptive statistics for the POI rate estimates.

Results

High POI rate was seen in males (1.14%), and adults (36-50 years, 1.25%) with 2.65 times (95% CI 1.69-4.12) higher odds compared to young adults. POI rate in ERCP inpatients varied widely according to geographic region: higher in the West (1.46%) and Northeast (1.20%) and lowest in the Midwest (0.70%). As per the hospital characteristics, inpatients in public (1.26%), urban teaching (1.39%) and small bed-size (1.27%) hospitals had higher POI rates. ERCP inpatients with POI had higher odds of association with comorbid HIV infection (OR 1.55, 95% CI 1.13-2.12) and diabetes (OR 1.43, 95% CI 1.09-1.85). ERCP inpatients with POI had a significantly longer length of stay (LOS) by 5.2 days and higher total charges by USD 53,966 than inpatients without POI.

Conclusions

POI is associated with acute inpatient care, with longer hospitalization stays and higher costs, leading to increased healthcare burdens. The main goal is to identify the risk factors and to prevent POI with prophylactic antibiotics.

## Introduction

Since its introduction in 1968, endoscopic retrograde cholangiopancreatography (ERCP) has become a commonly performed endoscopic procedure. The diagnostic and therapeutic utility of ERCP has been well demonstrated for a variety of disorders, including the management of choledocholithiasis, biliary and pancreatic neoplasms, and the postoperative management of biliary perioperative complications [1]. ERCP is the primary therapeutic procedure for the treatment of diseases that affect the biliary tree and pancreatic duct [2]. While the therapeutic success rate of ERCP is high, the procedure can cause complications, such as acute pancreatitis, bleeding, and perforation [3]. The significance of post-ERCP complications reveals the necessity of their avoidance by adopting additional measures if risk factors are identified [4].

Post-ERCP infections are reported to occur in less than five percent of all interventions [5]. High hygienic standards for the intervention itself and thorough disinfection and storage of endoscope and endoscopic devices have fundamentally attributed to this low infectious rate [6]. Procedural improvements such as endoscopic decompression by biliary stents and immediate placement of percutaneous biliary drainage if endoscopic drainage is not possible, represent further strategies to reduce the incidence of ERCP-related infectious complications [7].

Complications related to ERCP is an important issue as the failure to restore adequate drainage after injection of contrast media into obstructed bile tracts during ERCP still represents the major risk factor for post-ERCP infection [8]. Our first objective in this study is to provide a descriptive overview of the postoperative infection (POI) following ECRP procedure during hospitalization and determine the rates of POI stratified by patients’ demographic characteristics and hospitals’ characteristics in the United States, and associated comorbid risk factors. The second objective is to evaluate the demographic and comorbid risk factors associated with POI in ERCP inpatients, and its impact on the length of stay (LOS) and total charges.

## Materials and methods

We conducted a retrospective cross-sectional study using the data from 2012 to 2014 Nationwide Inpatient Sample (NIS) of the Healthcare Cost and Utilization Project (HCUP) [9]. The NIS is the largest publicly available nationwide inpatient database from a sampling of 4,411 hospitals across 45 states representing a pool of about 20% of the US hospitals [9].

Patient variables included in this study are age, gender, race, median household income, primary payer, diagnosis, principal procedure, LOS, and total charges [10]. Hospital variables drawn from the American Hospital Association (AHA) Annual Survey of Hospitals included ownership, bed size, location/teaching status, and region [10]. The region was summarized based upon U.S. Census Bureau and AHA Annual Survey of Hospitals [10].

We included adult inpatients (18 to 65 years) who had a principal procedure of ERCP (ICD-9 procedure code, 51.10). These inpatients were divided into groups, with and without POI (ICD-9 diagnosis code, 998.59). We used cross-tabulations to obtain a descriptive overview of the rate of POI in ERCP inpatients stratified by their sociodemographic characteristics as well as hospital characteristics. Differences in continuous variables like LOS and total charges between POI and non-POI groups were measured using the independent sample t-test. Risk factors that may predispose POI in ERCP inpatients were identified by ICD-9 diagnosis or clinical classification software (CCS) codes namely, bile duct perforation (ICD-9 576.3), obstruction of the stent (ICD-9 576.2), diabetes (CCS code 49) and human immunodeficiency virus (HIV) infection (CCS code 5). Logistic regression model adjusted for age, gender, and the race were conducted to estimate the odds ratio (OR) of these risk factors for POI in ERCP inpatients.

We used SPSS version 25 (IBM Corporation, Armonk, New York) to analyze the NIS data and apply discharge weights [10] to attain a nationally representative sample. We used a logistic regression model and descriptive statistics with 95% confidence intervals (CI) for the POI rate estimates, and the statistical significance was set at P-value < 0.05.

## Results

From 2012 to 2014, a total of 28,525 ERCP as a primary procedure were conducted with only 300 inpatients with co-diagnoses of POI (1.05%). Relatively high rates of POI were seen in adults (36-50 years, 1.25%) with 2.65 times (95% CI 1.69 - 4.12) higher odds of association compared to young adults (18-35 years). As per other race/ethnicity and gender, we did not find statistically significant differences in POI with the highest rate seen in male (1.14%) and white (1.27%). In the adjusted regression model, Native Americans have 4.7-fold higher odds (95% CI 2.44 - 0.19) for POI compared to white. A higher rate of POI was observed in Medicare (1.22%) and private insurance (1.08%) beneficiaries.

POI rate in ERCP inpatients was diverse broadly by geographic region in the US. The rate of POI was higher in the West (1.46%) and the Northeast (1.20%) followed by the South (0.94%) and lowest in the Midwest (0.70%). As per the hospital characteristics, patients in public (1.26%), urban teaching (1.39%) and small bed-size (1.27%) hospitals had higher rates of POI compared to their respective counterparts. The rate of POI in ERCP inpatients varied extensively across sociodemographic and hospital strata as shown in Table 1.

**Table 1:** Sociodemographic and hospital characteristics, and comorbid risk factors of ERCP inpatients and those with postoperative infection The proportion between total ERCP inpatients and inpatients with postoperative infections were obtained using cross-tabulation. POI: postoperative infection; HIV: human immunodeficiency virus

Characteristic	Total (N= 28525)	POI (N= 300)	POI rate	P-value
Age at admission (in years)				
18 – 35	5670	25	0.44%	<0.001
36 – 50	8375	105	1.25%	
51 – 65	14480	170	1.17%	
Gender				
Male	12280	140	1.14%	0.203
Female	16245	160	0.98%	
Race				
White	16425	210	1.27%	0.425
Black	3915	20	0.51%	
Hispanic	4580	30	0.65%	
Asian	830	0	0%	
Native American	170	10	5.88%	
Other	1185	20	1.69%	
Median household income, in percentile				
0-25^th^	8215	45	0.55%	<0.001
26^th^-50^th^	7425	105	1.41%	
51^st^-75^th^	6775	60	0.89%	
76^th^-100^th^	5315	75	1.41%	
Primary payer/insurance				
Medicare	4900	60	1.22%	0.884
Medicaid	6345	65	1.02%	
Private insurance	12950	140	1.08%	
Uninsured	4255	35	0.82%	
Hospital ownership				
Public	4370	55	1.26%	0.154
Private, non-profit	20965	215	1.02%	
Private, for-profit	3190	30	0.94%	
Hospital location and teaching status				
Rural, non-teaching	1320	10	0.78%	<0.001
Urban, non-teaching	8125	25	0.31%	
Urban, teaching	19080	265	1.39%	
Hospital bed size				
Small	3160	40	1.27%	0.121
Medium	7150	80	1.12%	
Large	18215	180	0.99%	
Hospital region				
Northeast	5845	70	1.20%	0.079
Midwest	6480	45	0.70%	
South	10055	95	0.94%	
West	6145	90	1.46%	
Comorbid risk factors				
Diabetes	6070	85	1.40%	0.003
HIV infection	3945	55	1.40%	0.023
Bile duct perforation	20	0	0%	0.645
Obstruction of stent	6715	55	0.82	0.033

In logistic regression model adjusted for demographics as shown in Table 2, ERCP inpatients with POI had higher odds of association with comorbid HIV infection (OR 1.55, 95% CI 1.13 - 2.12) and diabetes (OR 1.43, 95% CI 1.09 - 1.85). On the contrary, bile duct perforation and obstruction of the stent were not associated with higher odds for POI after controlling for other risk factors. 

**Table 2:** Predictors of postoperative infections in ERCP inpatients The odds for the ERCP inpatients with postoperative infections were obtained using logistic regression model and were significant with P-value ≤0.05 at 95% confidence interval. HIV: human immunodeficiency virus

Characteristic	Odds Ratio	95% Confidence Interval		P-value
		Lower	Upper	
Age at admission (in years)				
18 – 35	Reference			
36 – 50	2.65	1.69	4.12	<0.001
51 – 65	2.26	1.45	3.50	<0.001
Gender				
Male	Reference			
Female	0.94	0.75	1.19	0.636
Race				
White	Reference			
Black	0.39	0.24	0.61	<0.01
Hispanic	0.54	0.37	0.80	0.159
Asian	<0.01	<0.01		0.990
Native American	4.74	2.44	9.19	<0.01
Other	1.28	0.81	2.04	0.292
Comorbid risk factors				
None	Reference			
Diabetes	1.43	1.09	1.85	0.008
HIV infection	1.55	1.13	2.12	0.006
Bile duct perforation	<0.01	<0.01		0.998
Obstruction of stent	0.61	0.45	0.83	0.002

Table 3 shows the results of the independent sample T-test. ERCP with POI inpatients had a significantly longer LOS by 5.2 days (95% CI -0.49 to -5.98) and higher total charges by USD 53,966 (95% CI -61,404 to -46,527) than inpatients without POI.

**Table 3:** Differences in length of stay and total charges in ERCP inpatients with postoperative infections POI: postoperative infections; CI: confidence interval

Variable	Mean (Standard deviation)		Mean difference		
	Non-POI	POI	Estimate	95% CI	P-value
Length of stay, days	5.8 (6.66)	11.0 (8.41)	-5.22	-0.49 to -5.98	<0.001
Total charges, USD	53,554 (63,909)	107,520 (124,100)	-53,966	-61,404 to -46,527	<0.001

## Discussion

From our study, the majority of risk factors for the post-ERCP infection were amongst white males with a median age of 42 (interquartile range 35 to 50 years). The exact source of infection has not been demonstrated, but pancreatic sepsis or abscess can be caused by the introduction of dangerous materials into a stagnant duct system, with the endoscopic cannula or contrast medium [11]. Although there is no absolute evidence for the effectiveness of routine antibiotic prophylaxis on the frequency of septic complication after ERCP, the rate of bacteremia seems to decrease significantly. After ERCP, there is often temporary abdominal discomfort, transient fever, and hyperamylasemia [12].

According to Kullman et al., the occurrence of POI is associated with therapeutic ERCP, but due to a small sample population, there were not able to reach statistically significant differences between both diagnostic and therapeutic procedures [11]. Mingmei et al. suggested that POI occurs irrespective of diagnostic and therapeutic ERCP procedure because they excluded all the infections that occurred preoperatively [13]. Its clinical features, management, and the outcome are poorly characterized in the literature [12]. In a recent study, (200 procedures in 22 months) the reported rate of POI was less than 5%, with a tendency to reduce with increased experience of the practitioner [12]. In our study, from a total of 28,525 ERCP performed, only 300 inpatients were diagnosed with comorbid POI.

POI was seen more in diabetes due to hyperglycemia, which causes increased virulence of infectious microorganisms and apoptosis of polymorphonuclear neutrophils. Diabetic patients have decreased neutrophil function, decreased T-lymphocytes response, and humoral immunity disorders [14]. According to our study, post-ERCP infection is more common in Native Americans as they live in rural areas and high prevalence of hospitalization because they have greater exposure to environmental risk factors for infection. This can be overcome if they improve access to water, sanitation, and alternative sources of heat in rural areas [15].

The most prevalent isolates were* E. faecium* and *E. coli *seen by the studies conducted by Rerknimitr et al. and Thosani et al. in patients undergoing ERCP [16-17]. Prophylactic antibiotics are the most effective prevention against infections from gram-negative bacteria and *Enterococcus spp.* Guidelines by the American Society for Gastrointestinal Endoscopy (ASGE) and the British Society of Gastroenterology Endoscopy advocate prevention for patients with biliary obstruction [18-19], whereas the European Society of Gastrointestinal Endoscopy [20] recommends prophylaxis for every type of therapeutic ERCP. ASGE guidelines currently recommend that antibiotic prophylaxis should be considered before an ERCP in patients with known or suspected biliary obstruction [18] in which there is a possibility that complete drainage may not be achieved, such as in patients with a hilar stricture and primary sclerosing cholangitis [11]. When biliary drainage is incomplete despite an ERCP, continuation of antibiotics after the procedure is recommended. Antibiotics that cover biliary flora, such as enteric gram-negative organisms and enterococci, should be used [18].

Preoperative admission to an intensive care unit (ICU) increased the odds of POI as compared to hospital admission outside of an ICU. Being admitted at least one day before procedure compared to the same-day procedure was associated with a significant increase in the odds of POI. There was no significant independent association between preoperative length of stay and risk of POI while POI and postoperative length of stay were significantly associated [21]. The main goal is to identify the risk factors and to prevent POI with antibiotics like intravenous moxifloxacin (400mg/day) or ceftriaxone (2g/day) administered for three days or more if the patient developed signs and symptoms of cholangitis or septicemia [22].

This study has a few limitations. Some cases of bacteremia would have gone undetected because of a lack of blood culture results during hospitalization. Also, there might be under-reporting of co-diagnoses of POI during discharge diagnoses coding inpatient records. Another issue is that our study monitored all ERCP cases during hospitalization, but post-discharge surveillance was not carried out. Post-discharge monitoring could be performed to identify POI that was not reported and documented in our study sample.

## Conclusions

The risk factors for post-ERCP infection are middle-aged adults (36 to 50 years), Native American, immunodeficiency, and diabetes. The detailed epidemiological data for infections after ERCP in US hospitals may improve our understanding of the risk factors of post-ERCP infections and potential prevention and treatment measures. Our study indicates that the incidence of post-ERCP infection is high among these high-risk patients, and these enables the clinicians to consider these predictors when they start antibiotic prophylaxis. It is important to manage post-ERCP infections as they increase the need for acute inpatient care due to longer LOS and higher total charges compared to inpatients without postoperative infections.
